# Improving clinical decision-making in psychiatry: implementation of digital phenotyping could mitigate the influence of patient’s and practitioner’s individual cognitive biases

**DOI:** 10.1080/19585969.2022.2042165

**Published:** 2022-06-01

**Authors:** Stéphane Mouchabac, Ismael Conejero, Camille Lakhlifi, Ilyass Msellek, Leo Malandain, Vladimir Adrien, Florian Ferreri, Bruno Millet, Olivier Bonnot, Alexis Bourla, Redwan Maatoug

**Affiliations:** a Department of Psychiatry, Hôpital Saint-Antoine, Sorbonne Université, AP-HP, Paris, France; b Sorbonne Université, Hôpital de la Pitié Salpêtrière, iCRIN (Infrastructure for Clinical Research In Neurosciences), Brain Institute (ICM), INSERM, CNRS, Paris, France; c Department of Psychiatry, CHU Nîmes, University of Montpellier, Nîmes, France; d Inserm, Unit 1061 “Neuropsychiatry: Epidemiological and Clinical Research”, Montpellier, France; e PICNIC lab (Physiological investigation of clinically normal and impaired cognition), Institut du Cerveau - Paris Brain Institute - ICM, Inserm, CNRS, APHP, Hôpital de la Pitié Salpêtrière, Université de Paris, Sorbonne Université, Paris, France; f University Hospital Cochin (site Tarnier), Paris, France; g Department of Child and Adolescent Psychiatry, CHU de Nantes, Nantes, France; h Pays de la Loire Psychology Laboratory, Nantes, France; i Jeanne d'Arc Hospital, INICEA Korian, Saint-Mandé, France

**Keywords:** Digital phenotyping, cognitive bias, medical decision making

## Abstract

High stake clinical choices in psychiatry can be impacted by external irrelevant factors. A strong understanding of the cognitive and behavioural mechanisms involved in clinical reasoning and decision-making is fundamental in improving healthcare quality. Indeed, the decision in clinical practice can be influenced by errors or approximations which can affect the diagnosis and, by extension, the prognosis: human factors are responsible for a significant proportion of medical errors, often of cognitive origin. Both patient’s and clinician’s cognitive biases can affect decision-making procedures at different time points. From the patient’s point of view, the quality of explicit symptoms and data reported to the psychiatrist might be affected by cognitive biases affecting attention, perception or memory. From the clinician’s point of view, a variety of reasoning and decision-making pitfalls might affect the interpretation of information provided by the patient. As personal technology becomes increasingly embedded in human lives, a new concept called digital phenotyping is based on the idea of collecting real-time markers of human behaviour in order to determine the ‘digital signature of a pathology’. Indeed, this strategy relies on the assumption that behaviours are ‘quantifiable’ from data extracted and analysed through connected tools (smartphone, digital sensors and wearable devices) to deduce an ‘e-semiology’. In this article, we postulate that implementing digital phenotyping could improve clinical reasoning and decision-making outcomes by mitigating the influence of patient’s and practitioner’s individual cognitive biases.

## Clinical psychiatry: A peculiar decision-making context

Doctors can be seen as detectives: together with the victim (the patient) and the witnesses (his/her relatives), they collect pieces of evidence to identify the culprit (the diagnosis of a disease) and take the necessary measures (establish a treatment plan). In this course of action, various cognitive processes are being recruited, both in the patient and the practitioner (Elstein and Schwartz 2002): information perception, selection and interpretation in order to make a decision. Such clinical reasoning process can be based on several possible models: the inductive, the abductive and the hypothetico-deductive models.

The so-called ‘inductive’ reasoning model is grounded on the patient's complaint: the problem is drawn from clinical data that the practitioner interprets, compares or even simplifies. This strategy therefore leads to a construction of the problem oriented towards a solution.

The abductive model is rather a strategy for experienced doctors as it relies on a high number of similar situations already encountered in practice. Psychiatrists can browse through clinical databases structured around prototypes (average typical case of a situation), from which they can generate hypotheses, from automatic analogy. Therefore, abduction allows to identify a cause, which would be the most probable in the face of observed facts, and to formulate the hypothesis that the observed symptoms probably result from this cause.

Lastly, the ‘hypothetico-deductive’ model aims at testing hypotheses in order to generate one or more solutions to the problem. One must then collect data to refute or support the hypotheses.

Clinical reasoning can be based on one mode exclusively or combine several strategies (Marewski and Gigerenzer 2012). In fact, many parameters will influence the choice of a process, such as the complexity of the clinical situation, the level of experience (Norman et al. 2007) of the practitioner or his own beliefs.

Beyond the theory of decision-making in psychiatry: the reality. High stake clinical choices can be impacted by external factors such as the patient’s or practitioner’s fatigue, cognitive and emotional loads, stress… What is more, uncertainty, which scientifically corresponds to a margin of imprecision, directly affects clinical decision-making, by limiting our ability to produce accurate predictions. Hall (2002) proposes 3 major situational categories of uncertainty in medicine. The first arises from the lack of information to draw our hypotheses: this category is therefore technical. The second involves the nature of the relationship with patients, in particular when the patient’s own wishes are unknown from the doctor and when he/she is unable to participate in the decision-making process. Finally, a third category is directly linked to the way our brain works and states that our choices are never certain as to their potential effects.

Magnavita (2016) argues that there are five pillars of effective decision-making in psychiatry or psychology: (a) access to high-quality empirical evidence: In the rather young discipline of psychiatry, it must be recognised that the level of evidence is still average. Therefore, we do not always have the necessary information to make optimal decisions; (b) development of clinical expertise: Today, information is accessible to everyone on the Internet, but more specific skills are needed to transform information into useful insight for patient care. Even if the ‘expert’ is better suited than the average person to achieve this practical integration, specific factors can interfere with the expertise. For many, psychiatry is regarded as not scientific and its models are accused of being obsolete; (c) use of sound theoretical concepts: Theories in psychiatry evolve in real-time along with discoveries, which leads to new theories that need to be tested for their potential utility; (d) inclusion of ethical considerations, as our biases influence our decisions: An ethical framework can counterbalance the risk of their impact on complex clinical situations; (e) Study of the foundations of decision theory. As some authors have suggested, knowing the basics and associated concepts is essential.

## Our challenge: Mitigating the impacts of cognitive biases

As highlighted in the two last pillars of effective decision-making in psychiatry and psychology, a strong understanding of the cognitive and behavioural mechanisms involved in clinical reasoning and decision-making are fundamental for ethical considerations (Ludolph and Schulz 2018). Indeed, the decision in clinical practice can be accompanied by errors or approximations which can affect the diagnosis and, by extension, the prognosis. For instance, the study performed by Hatfield et al. (2009) illustrates the limitation of isolated clinical judgement in monitoring psychiatric symptoms in 70 patients showing worsening of their disorder. Increased severity was detected by classical clinical examination in only 21% of patients. These results raise the difficulty to detect the evolution of patients’ clinical state in specific situations and the need to use more objective data to complete the evaluation. Human factors are responsible for a significant proportion of medical errors (Kassirer and Kopelman 1989; Graber et al. 2005; Singh et al. 2013), most often of cognitive origin (Kassirer and Kopelman 1989; Graber et al. 2005; Singh et al. 2013; Zwaan and Singh 2015). They can appear at all levels of the clinical reflection process, from information collection to the therapeutic choice and its validation, and might be due to various constraints, both in patient’s and practitioner’s individual and social cognition: limited attention, partial perception, bounded memory, biased reasoning and collective phenomena (Doherty and Carroll 2020). We know for instance that the necessary amount of data to be processed in psychiatry exceeds the clinician's real-time analysis capacities. Among these constraints, cognitive biases have been recently extensively studied in the medical context, but their origin is not always discussed in the resulting publications (Croskerry 2003; Klein 2005; Croskerry et al. 2013; Saposnik et al. 2016; O'Sullivan and Schofield 2018).

Along the thousands of years of our species evolution, hominids have acquired cognitive skills to adapt to their environment. These aptitudes are optimised to solve the problems that our ancestors encountered in order to survive and reproduce. These mental shortcuts are called ‘heuristics.’ Their goal is to find efficient and rapid solutions to a variety of situations while saving mental energy. In the original hunter-gatherer environment, these cognitive tools improved our analytical and reaction skills, but in our complex modern world, they sometimes become unconscious relics that drive us to make ‘irrational’ decisions. When heuristics are misused and trigger non-optimal choices, they are characterised as ‘cognitive biases’. They were first studied in the early 1970s (Tversky and Kahneman 1974) to understand the mechanisms that could explain certain irrational decisions made in the economic sector, and the authors then extended their field of research to the question of human rationality in the broad sense. In order to explore this topic and better understand and describe the foundations of decision-making under uncertainty, Kahneman and Tversky proposed a theoretical framework (Kahneman 2011) composed of two components. The first, called ‘system 1’ (or rapid system), is mostly automatic, non-conscious, inexpensive in cognitive resources and optimised for the most usual decisions. The other component, called ‘system 2’ (or slow system), calls on conscious processes, is not dedicated to routine tasks and is more costly on the cognitive level. Albeit criticized (Melnikoff and Bargh 2018), this unperfect model is useful to better apprehend and decrypt the strategies that might be recruited in different situations for clinical reasoning and decision-making. In theory, when we use evidence-based medical reasoning or decision support systems, system 2 takes the lead. But sometimes, especially in the face of uncertainty, system 1 can “hack” our reasoning process that in turn relies on automatisms or intuitions: in these conditions, heuristics are privileged and biases can distort the ultimate choice (Norman et al. 2014, 2017). The two major products of clinical decision-making are diagnoses and treatment plans. If the first is correct, the second has a greater chance of being correct too. Surprisingly we do not make correct diagnoses as often as we think. The diagnostic failure rate is estimated to be 10% to 15%, as Pat Croskerry claims (Croskerry 2013). Cognitive biases can affect diagnosing procedures at different time points and from different perspectives. From the patient’s point of view, the quality of explicit symptoms and data reported to the psychiatrist might be affected his/her biases affecting attention, perception and memory. These cognitive mechanisms might themselves be impacted by other factors such as the level of fatigue or hunger, the patient’s mood or emotional state, the weather… From the practitioner’s perspective, the relevance of the diagnosis outcome built on this uncomplete and biased ground might also be influenced by the cognitive constraints previously mentioned, in addition to reasoning pitfalls due to cognitive biases (Mamede et al. 2014; Blumenthal-Barby and Krieger 2015; Saposnik et al. 2016; FitzGerald and Hurst 2017; O'Sullivan and Schofield 2018; Featherston et al. 2020) (for examples, see Table 1).

**Table 1.** Classification of the most common cognitive biases in medicine.

**Anchoring bias**	Tendency to focus on a first impression or on the first information received to form an opinion about a number, a person, an event… This judgmental bias can prevent important information received later to be taken into account.	Selection
**Ascertainment**	Tendency to selectively analyse clinical data in the light of prior expectations or beliefs (belief bias). This bias can impact the interpretation of new information resulting from precise surveillance or screening of certain symptoms.	Selection
**Availability bias**	Tendency to form an opinion based on the most recent and readily available information in one’s mind, considered more likely. For example, for an opinion on a treatment, we remember the last few patients rather than a series of 100.	Selection
**Base-rate neglect**	Type of error due to poor knowledge of disease incidence rates, either by underestimating or by overestimating the occurrence of a diagnosis.	Selection
**Confirmation bias**	Tendency to select and interpret information confirming a clinical intuition or a priori diagnosis, and to neglect information that contradicts or invalidates this intuition.	Selection
**Diagnosis momentum**	Diagnosis or treatment plans established by previous clinicians are rarely questioned by new practitioners and stick to the patient. This phenomenon can prevent considering new options and enhancing the diagnosis or provided healthcare.	Selection
**Illusory correlation**	Tendency to infer causation relationships between correlated but independent events.	Selection
**Premature closure**	Tendency to stop reasoning, evaluating or looking for a better diagnosis or treatment alternative after finding a suitable enough option (close to ‘satisfaction search bias’).	Selection
**Primacy effect**	Mnemonic bias, tendency to remember and consider more the first information out of a list of equal importance.	Selection
**Recency effect**	Mnemonic bias, tendency to remember and consider more the most recent information (received last), for example the last words of a clinical interview or the last symptoms of a list.	Selection
**Unpacking principle bias**	Type of error occurring when not all the necessary information were requested to make an objective judgement. The risk would be, for example, to omit information that would allow a differential diagnosis.	Selection
**Affect bias**	When decisions are made in a context where the immediate emotions are strong and can influence our choices.	Process
**Ambiguity or risk aversion**	Type of bias describing the tendency to favour choices with known risks and associated probabilities rather than ambiguous or uncertain options.	Process
**Commission bias**	Tendency to favour action over inaction, even when inaction would be more rational. It can result in overprescription.	Process
**Default bias or status quo bias**	Tendency to stick to the default option and avoid changes. The cost of change in terms of cognitive effort is automatically considered too great and one continues to behave in the same way.	Process
**Framing bias**	The perception of a situation can be influenced by the way options are being presented (formulation with different numerical presentations, or with positive or negative connotations…).	Process
**Information bias**	This bias translates into errors in the collection of information, for example during an interview: it can be a failure to observe, a misclassification or organisation of data, or errors in memory recall during synthesis.	Process
**Loss aversion**	Tendency to be more sensitive to the loss of a certain amount of resources (cognitive effort, time, money…) than to the gain of the same amount of resources, resulting in choices that tend to avoid losses rather than attempt gain.	Process
**Omission bias**	Tendency to favour inaction or to avoid difficult issues over action (‘wait and see’). It affects self-doubting clinicians.	Process
**Outcome bias**	Tendency to focus on the outcome of the decision rather than the information to be interpreted to make a relevant decision. This bias is more common among clinicians with lower self-confidence and can lead to an incorrect diagnosis.	Process
**Overconfidence**	Tendency to think that our knowledge or skills are greater than they actually are. The confidence miscalibration can result in non-optimal therapeutic actions and choices.	Process
**Representativeness restraint bias**	Tendency to rely on the ‘frequency argument,’ i.e., to favour the most common hypotheses and not to mention the rarer ones. It is a restriction of thought that prevents a broader questioning of a clinical situation.	Process
**Retrospective prejudice**	When the result of a situation is known, it can influence the way in which we perceive the preceding events as we forget the uncertainty we were facing at that time, and lead to fallacious reconstruction (‘we are remaking history’). It can prevent learning and lead to the repetition of error.	Process
**Self-served bias**	Tendency to reduce the analysis of clinical data and the diagnosis to one's own point of view. It affects communication between the different parties (physician, patients, and other stakeholders).	Process
**Sunk cost fallacy**	Tendency, when one has already invested a lot of resources (time, energy or money) in a project or an action that seems to have little chance of succeeding, to continue investing although it is doomed to failure. In medicine, it is a question of pursuing an ineffective strategy, for example.	Process
**Bandwagon effect**	Tendency to conform and reproduce a behaviour or an attitude just to act as others do.	Social
**Fundamental attribution error**	While making judgments about people’s behaviour, it's the tendency to overemphasise dispositional factors or personality-based explanations and underestimate situational ones. The consequence is the risk of making incorrect judgments, discounting reasons that might have contributed to their observed behaviour.	Social
**Stereotyping**	Tendency to infer characteristics about an individual based on the group in which we categorised him/her. This can result in a wrong diagnosis solely based on our belief that the patient belongs to a certain group with a typical disease.	Social

Several classifications of cognitive biases co-exist. Here, we propose to group them according to when they might impact clinical reasoning or decision-making in psychiatry: the selection of relevant information to form an opinion, the processing of these selected pieces of information, and social biases that are errors generated from our social brain (ACAPS model) and can influence the processing of information based on the nature of relationships.

Knowing these biases and the associated risks is therefore crucial for improving medical decisions, especially in psychiatry, where uncertainty can be significant. Along with promoting more personalised support to each patient, mitigating the impacts of cognitive biases on psychiatric healthcare is a challenge that needs to be addressed. While some authors (Gigerenzer and Brighton 2009; Marewski and Gigerenzer 2012) questioning the relevance of the concept of cognitive biases suggest that training heuristics through experience in order to refine them might be a way to optimise medical decision-making under uncertainty (Gigerenzer and Kurzenhäuser 2005; Wegwarth et al. 2009; Marewski and Gigerenzer 2012), new technologies potentially offer new solutions that must therefore be evaluated.

## Digital phenotyping: An advanced tool to objectivise diagnosis

In 1982, the evolutionary biologist Richard Dawkins stressed that many delineations of phenotypes are arbitrary and introduced the concept of the ‘extended phenotype’: the idea that phenotypes should not be limited just to biological processes, such as protein biosynthesis or tissue growth, but extended to include all effects that a gene has on its environment, may it be inside or outside of the body of the individual organism. Animals and humans can modify their environment, and these modifications and associated behaviours are expressions of one’s genome and, thus, part of their extended phenotype. Dawkins cites dam-building by beavers as an example of the beaver’s extended phenotype.

As personal technology becomes increasingly embedded in human lives, a new concept called digital phenotyping was defined in 2015 by Jain et al. (2015) and shortly after by John Torous in the field of psychiatry. Digital phenotyping is a concept based on the idea of collecting real-time data and markers of human behaviour to determine the ‘digital signature of a pathology.’ From a theoretical point of view, it is assumed that behaviours are ‘quantifiable’ from data extracted and analysed through connected tools (smartphone, digital sensors and wearable devices) to deduce an ‘e-semiology.’

**Figure 1.**
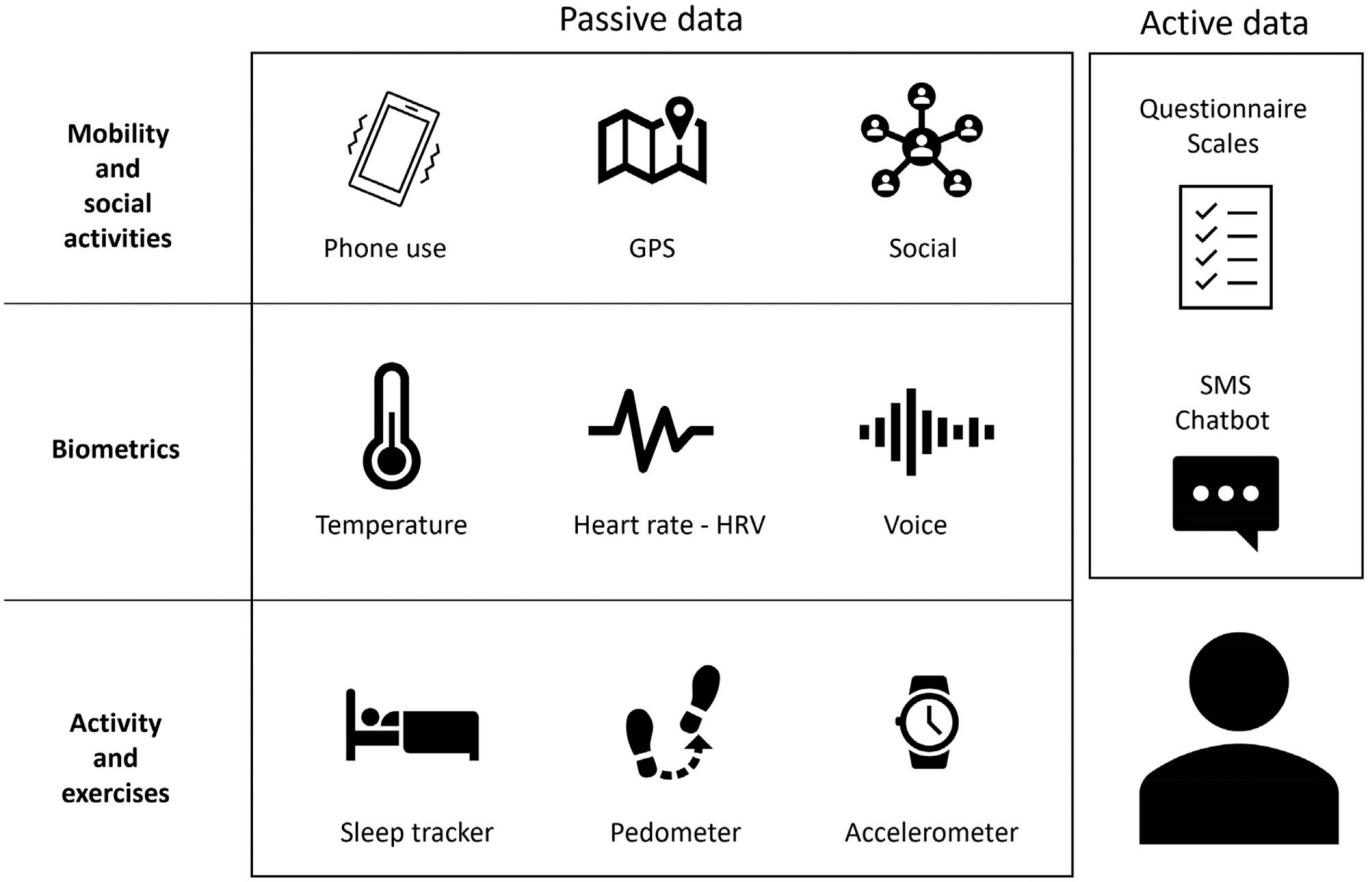
Digital phenotype overview.

As shown in Figure 1, two types of data are taken into account. So-called ‘passive’ data are collected automatically, in real-time, without requiring any input from the user. Passive data collection relies on tools such as the accelerometer and GPS, for example, to search for psychomotor retardation or on the contrary motor agitation. More precisely, changes in the GPS coordinates compared to the usual travel behaviour (decrease in activity, increase in time spent at home or, conversely, chaotic journeys in unusual places) could signal pathological behaviours. Mobile phone-based sensing software allows us to measure heart rate, heart rate variability (HRV), galvanic skin conductance or blood pressure. These indicators are considered as potential biomarkers of certain pathologies in psychiatry (Insel 2017; Rodriguez-Villa et al. 2020). The time spent in front of a screen, the number of calls and the hours of use provide good indicators of psychosocial functioning. Finally, the analysis of language (verbal and paraverbal) and even emotional arousal provide discriminating data on the valence of messages during social interactions and are indicators of the user’s mood and mental state.

On the other hand, it is essential to supplement this information by collecting subjective data, essential in psychiatry. In this perspective, the analysis of so-called ‘active’ data involves the patient consciously with self-reporting. The momentary ecological assessment is the most suitable method, as the patient may be called upon regularly at scheduled times during the day or in response to variations in their ‘passive’ parameters (Connolly et al. 2021). A psychometric scale specific to the pathology studied can be proposed, or a visual analog scale for more transnosographic symptoms (Figure 1).

The use of machine-learning allows this digital signature to be exploited at an individual level (intra-subject variations) but also by comparison to larger databases including other patients, to obtain predictive information. John Torous suggests that the criteria RDoC from NIMH, used to better define and understand the mechanism of mental illness, rely on digital phenotyping data for research and diagnostic improvement (Torous et al. 2017). The digital phenotype has been studied in many pathologies such as mood disorders, schizophrenia, anxiety disorders or even addiction (Ferreri et al. 2018; Jacobson et al. 2019).

A way to address our challenge of minimising the impact of patient’s and practitioner’s cognitive biases to optimise care decisions could therefore be to objectivise clinical data collection about the patient through digital phenotyping. In this conceptual paper, we propose to investigate and highlight the contribution of the digital phenotype in psychiatric clinical decision-making processes, particularly in the management and mitigation of the most frequently reported cognitive biases.

## Implications, limitations and perspectives

Conceptually, digital phenotype could support the diagnosis process by delivering personalised, objective and evidence-based observations or recommendations in real-time, both to clinicians and patients (Onnela 2021). To achieve this, such a system needs to collect and consider all the data about the patient – current complaints, physical findings, other co-morbid conditions, allergies, lab and imaging tests done over time. In short, the automated system has to take into consideration all the evidence clinicians usually use to establish a diagnosis or a treatment plan.

Once the data about the individual are gathered, they are compared to a large base of clinical data in order to search for matching patterns and predict outcomes (Torous et al. 2018). A differential diagnosis (the different possible diagnoses at the time of observation, in order of decreasing likelihood) can be created, further testing to better distinguish between the possibilities can be suggested, and a treatment plan can be proposed.

Clinical reasoning and decision-making outcomes can benefit in several ways from arising in the light of both active and passive collected data through digital phenotype.

First, as we mentioned earlier, the phase of clinical observation performed both by the clinician and the patient him/herself can be biased in many ways due to cognitive (attentional, perceptual, memory and reasoning) human constraints. Subjective explicit reports from patients can for instance suffer from the availability heuristic by relying only on immediately available stories or pieces of evidence in one’s mind, from the recency bias by referring to most recent experiences instead of a representative sample of events, or more simply from forgetfulness or situational factors (mood, fatigue…). For example, a patient will refer more frequently to his/her last three nights while reporting his/her sleep quality, rather than to a 15-day analysis of his sleep diary. The suitability of a hypnotic prescription can be impacted by the degree of precision of reported sleep quality.

A specific characteristic of passive data collection lies in the fact that it does not require any active input from the patient, enabling to reduce the influence of consciously reflecting or a posteriori analysing one’s experience. Indeed, explicitly reporting a feeling or an experience might in turn impact this very information. For instance, if a patient needs to hold a sleep diary, it might attract his/her attention on the moon phases: if he/she believes that full moon affects his/her sleep, he/she might end up having a night of poor quality by a self-fulfilling prophecy. Information collected and gathered repeatedly and continuously (the frequency can be adapted to the studied phenomenon), timely (at the moment they are being experienced by the patient, not as being remembered) and more objectively through digital phenotype can help the patient avoid a loss of valuable contextual elements and building a more precise picture of his/her symptoms to the psychiatrist. Thanks to all collected data, we can build large databases, compare them within and between subjects, identify trends over configurable durations, interpret them in the light of environmental data (social interactions, GPS) in order to interpret possible anomalies and make detailed medical decisions.

**Figure 2.**
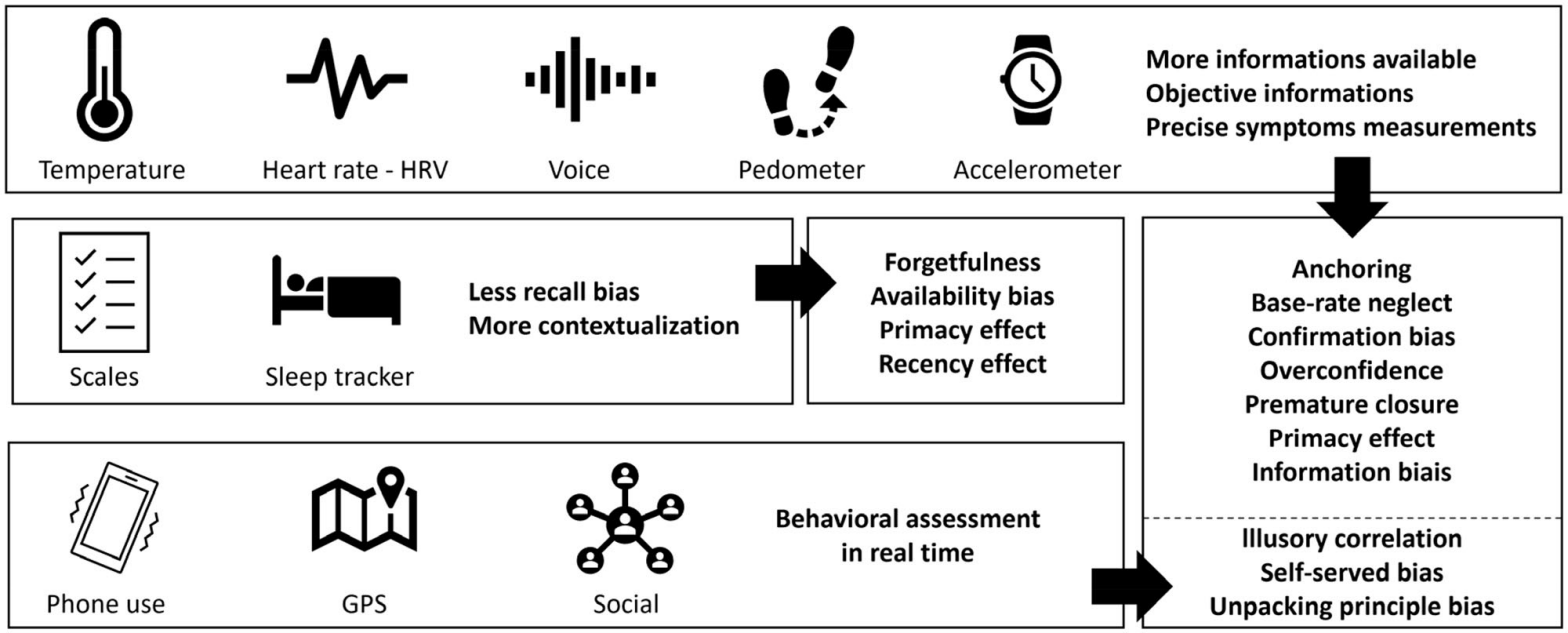
Digital phenotype can mitigate the impact of cognitive bias in various ways.

During the so-called orientation phase, the clinician might be influenced by his culture, his theoretical knowledge and his practical experience: many biases can affect his ability to analyse and synthesise the observed information at that stage. The practitioner might for example be affected by the confirmation bias and tend to only perceive and take into account the evidence confirming a prior opinion (in our case, an idea of diagnosis), omitting information conflicting with this prior opinion. The anchoring bias could drive the clinician to focus too much attention on the first information or symptoms reported by the patient, or to stick by status quo to a diagnosis suggested by a colleague (diagnosis momentum). The way the patient reports his/her feelings could also impact the psychiatrist’s analysis through the framing effect (for more examples, see Table 1). The parameterisation of the technical collection of information (sampling frequency, number of parameters involved), coupled with machine-learning algorithms could make it possible to more precisely distinguish diseases signatures and to limit these errors (Figure 2).

The decision phase often requires coping with uncertainty as well as data complexity and ambiguity. While these processes are sometimes performed ina controlled and rigorous way (analysis and synthesis), they might as well rely on heuristics and rather be automatic. To reduce the effects of biases on these processes, researcher satisfaction or premature closure bias, for example, the available data set about the patient makes it possible to expose the clinician to objective elements related to his or her complaint, preventing him/her from ignoring this information.

Finally, cognitive biases represent a technical challenge, as improving the quality and objectivity of the observation and orientation phases does not prevent the psychiatrist from making errors in the subsequent reasoning step: decision-making. Aversions to loss and ambiguity can lead to non-optimal healthcare choices. The causality temptation with the illusory correlation remains a frequent risk and the self-served bias will affect the nature of the communication with the patient. To address these risks, solutions such as the addition of a clinical decision support system that can be based on AI are promising. These CDSSs must use active and passive data to deliver personalised strategies while relying on large databases and adjustable recommendations in real-time or on-demand.

After all, social biases (stereotyping, bandwagon effect) can influence the processing of information based on our relationships with people with whom we interact, due to specific social representations and perceptions of individuals or current social trends. They seem difficult to mitigate through technology. However, the amount of data collected can make it possible to determine specific digital signatures, which even if they refer to general categories, can still be analysed at an individual level. As the psychiatrist has this information, he/her can be assisted to review his judgement or stereotype. The interoperability of the data must be tailored to increase their chances of being considered by clinical decision protagonists and to avoid digital phenotype being considered as a clinical gadget.

As Onnela points out about passive data (Onnela and Rauch 2016), even if its collection is intended to be objective, it does not allow a constant understanding of psychiatric symptomatology. Certain clinical signs are indeed quite easy to measure, such as psychomotor retardation or agitation using GPS coordinates and the accelerometer. Meanwhile, for more complex symptoms involving several dimensions (for example cognitive disorders), several measures must be compared and be interpreted using more elaborate data processing (machine-learning). Labelling the data to be analysed also requires upstream expertise. Raw data and data already associated with symptoms and the way they are being processed will also depend on the a priori conceptions of developers and experts involved in designing these applications. In the same perspective, the large databases used to extract disease patterns has to be representative of the target patients’ population, in order to avoid reproducing biased data collection and interpretation at a larger scale. The analysis of missing data is a recurring problem in medical research. However, if digital phenotype is supposed to promote objective measurements, we cannot be sure that the raw data is being collected continuously. Technical constraints (battery) can appear as limiting factors. In addition, data capture and analysis must be done in context: a phone forgotten at home might for instance be interpreted as a nap and add noise to the analysis. What is more, although this effect might be reduced compared to subjective patient’s explicit reports, we cannot exclude that knowing that data are being actively and passively collected through digital phenotype does not in turn impacts the patient’s behaviour and habits.

Before the sophistication of the algorithms, the first limitation encountered with the implementation of digital phenotype in healthcare relates to the availability of data. Breaking down the institution-centric and provider-centric data silos is still work in progress in the medical context, although the situation is slowly evolving. Access to data is key, in order for digital phenotype learning to yield truly important insights.

Regardless of the promising support digital phenotype could provide to clinical reasoning and decision-making in psychiatry, several limitations need to be kept in mind and dealt with. First, the use of digital phenotype for objective data collection cannot be assimilated with a ‘debiasing’ intervention: this tool does not raise awareness of clinicians about the pitfalls they might encounter, but it rather helps create a context in which such occasions happen less frequently. Implementing digital phenotype does not eradicate cognitive biases, it minimises the situations in which they might impact information collection and processing. Second, one must keep in mind all the possible information that is not being collected through the digital phenotype and avoid having all his/her attention attracted on digital phenotype measurements only. Third, a major consideration lies in how to protect the individual private collected information: specialised and secured platforms need to be implemented in order to store and process data, and control for who can access them. Last but not least, the way (frequency, representation and framing) collected data will be presented both to the clinician and the patient has to be thought carefully and tailored in order to minimise the risk of misinterpretations. The illusory correlation bias could for example lead one or the other to a non-existent causation relationship between two pieces of information, in turn decreasing the quality of the diagnosis or treatment plan.

Within the next few years, medical data will likely be sufficiently aggregated to allow robust digital phenotype to be implemented. Indeed, the data collected for each subject will come both from the subject himself and also from doctors he has consulted and also from measurement tools (blood sample, imaging data …). This amount of data, will then, be compared to a large dataset of patients and healthy subjects in order to know if the subject has a higher chance to be sick (My 2021). It can be integrated into the tools clinicians and patients already use (EHRs, portals, etc.) to practice a personalised medicine (Mouchabac et al. 2021). Digital phenotype represents a robust opportunity for collecting objective information and providing feedback that could in turn promote learning and strengthen metacognitive skills, both for clinicians to gain experience and for patients to get to know themselves better!

## Conclusion

The real-time collection of passive data through digital phenotyping contributes to bypassing numerous biases, arising both from patient’s and practitioner’s individual and social cognition and impacting the quality of clinical choices. The systematic recording of some digital markers may for example help to counter information selection biases. In fact, the implementation of this technology allows to systematically collect data in real-time and over a longer and therefore more representative period, thus avoiding restraining data collection to the medical visit only. Hence, it may prevent memory biases from the patient him/herself, and also temper the effect of information (un)availability on the physician’ decision. Furthermore, the extensive quantification of multimodal measures (which can be combined at a time point through the digital device) may ensure exhaustivity of data collection preceding clinical decision-making. Therefore, it could allow counterbalancing anchoring and confirmation biases which affect the way the physician seeks relevant information. The implementation of algorithms defined a priori for data processing may maintain the analytic procedure apart from social and contextual influence, including stereotypes and halo effects. However, one must keep in mind that algorithms themselves are determined by humans, who may be likely to transfer their own biases in the machine. For this reason, automatic data processing may be considered with caution (In oration with Bourla et al. 2018, 2020).
